# Impact of COVID-19 on Mental Health: Sociodemographic Differences and the Moderating Effect of Religiosity

**DOI:** 10.3390/ijerph22101599

**Published:** 2025-10-21

**Authors:** Ivica Fotez, Zudi Osmani, Aleksandar Vcev, Lara Fotez, Darko Kotromanovic, Lucija Fotez, Mate Car, Gordana Horvat, Ivan Miskulin, Maja Miskulin

**Affiliations:** 1 Faculty of Dental Medicine and Health Osijek, Josip Juraj Strossmayer University of Osijek, 31000 Osijek, Croatia; ivica.fotez@vt.t-com.hr (I.F.); aleksandar.vcev@fdmz.hr (A.V.); 2 Faculty of Health Studies, University “VITEZ”, 72 270 Travnik, Bosnia and Herzegovina; zudi@bih.net.ba; 3 Institute for Public Health of Central Bosnia Canton, 72 270 Travnik, Bosnia and Herzegovina; 4 Health Centre Zagreb East, 10000 Zagreb, Croatia; fotezlara@gmail.com; 5 Faculty of Medicine Osijek, Josip Juraj Strossmayer University of Osijek, 31000 Osijek, Croatia; kotromanovic93@gmail.com (D.K.); maja.miskulin@mefos.hr (M.M.); 6 Oncology Clinic, University Hospital Center Osijek, 31000 Osijek, Croatia; 7 Institute for Emergency Medicine of Krapina-Zagorje County, 49000 Krapina, Croatia; lucija.fotez@gmail.com; 8 Center for Data-Driven Policy and Management, Catholic University of Croatia, 10000 Zagreb, Croatia; mate.car@unicath.hr; 9 Faculty of Law in Osijek, Josip Juraj Strossmayer University of Osijek, 31000 Osijek, Croatia; gordana.horvat@pravos.hr

**Keywords:** COVID-19 pandemic, mental health, sociodemographic factors, religiosity, survey

## Abstract

Background and Objectives: This research aimed to examine the impact of sociodemographic characteristics on mental health during the COVID-19 pandemic, with religiosity as a moderator. Materials and Methods: The cross-sectional study was conducted in family medicine clinics within the Primary Healthcare Center of Virovitica-Podravina County among 1131 participants, divided into 2 groups: RC (Recovered from COVID-19; N = 423) and NRC (Not Recovered from COVID-19; N = 708). To ensure clear differentiation, RC participants were defined as individuals with documented positive PCR results for SARS-CoV-2 (prior infection and clinical recovery), whereas NRC participants exhibited consistently negative PCR results and lacked any clinical history of the disease. Group allocation was rigorously based on the review of medical records and corresponding PCR documentation obtained both at the time of recruitment and retrospectively. All data were collected through a questionnaire from September 2022 to September 2023. Participants completed questionnaires measuring their sociodemographic characteristics (gender, age, education, and marital status), levels of depression, anxiety, stress, and level of religiosity. Results: Older participants were more prone to depression, whereas younger participants showed relatively better mental-health indicators. Sociodemographic characteristics were significantly associated with mental health during the pandemic. Religiosity was found to be a significant moderator in the relationship between sociodemographic characteristics and mental health. Individuals with higher levels of religiosity reported higher levels of depression and anxiety, suggesting that religiosity may act as a negative factor in times of crisis. Conclusions: Sociodemographic characteristics were significant predictors of mental health during the pandemic. Religiosity emerged as an important factor, particularly in moderating the relationship between sociodemographic characteristics and mental health. Further research is recommended to develop targeted interventions for vulnerable groups such as women, younger individuals, and those with lower incomes.

## 1. Introduction

The first case of infection with the COVID-19 virus was recorded in December 2019 in Wuhan, and since this virus appeared, it has spread throughout different countries of the world [1] and caused a large number of infections and deaths [2]. Precisely because of the aforementioned spread of the COVID-19 virus, the World Health Organization declared a global pandemic on 11 March 2020 [1]. According to data from the World Health Organization [3], by 18 May 2025, slightly less than 778 million cases of infection with this virus were recorded in the world, with 1,317,444 cases of infection recorded in Croatia. However, the evolution process of this virus is still unpredictable [4]. The clinical characteristics of the COVID-19 virus vary from asymptomatic conditions to severe respiratory distress [5]. However, this virus not only has physical effects on the human body. Namely, the spread of this virus has threatened global public health, and social systems have begun to break down, and in some countries, intensive care units were facing overcapacity. Most countries have implemented quarantine for the purpose of protection against the pandemic [4]. Thus, in order to prevent the spread of the virus, various measures were applied, such as closing schools and suspending production and commercial activities. The original intention of such measures was to protect people’s health, but they significantly affected people’s daily lives and their work activity, and although the guidelines for protecting the public from the virus were easy to follow, fear and prejudice greatly influenced the responses of the community in relation to the set guidelines. The pandemic has caused a high level of panic among people, which was illustrated by the example of empty shelves in stores, and the panic has influenced the seeking of medical help at the early onset of symptoms of infection with the COVID-19 virus [6].

The sudden emergence of the COVID-19 pandemic, coupled with its rapid spread, significantly impacted people’s lives [2]. Feelings of anxiety and insecurity are common in unfamiliar circumstances, particularly when new diseases arise whose progression and outcomes are unpredictable [6]. This prompted researchers to investigate the psychological effects of the pandemic, revealing that individuals have experienced depression, anxiety, and stress [2]. Previous epidemics, such as SARS, Ebola, and swine influenza, also resulted in negative consequences for mental health [7], including fatigue, a sense of social disconnection, stress, anxiety, decreased motivation, irritability, insomnia, and similar issues. Individuals respond differently when they perceive a situation or circumstance as threatening, which can lead to maladaptive behaviors, emotional distress, and defensive reactions such as anxiety, fear, frustration, anger, boredom, and depression [8].

Furthermore, individuals infected with the COVID-19 virus exhibited a range of neuropsychiatric symptoms, most commonly fatigue and depression, indicating that the virus may affect the central nervous system [9]. The phenomenon known as “headline stress disorder,” a characteristic of modern pandemics, refers to the intense emotional reactions that arise due to overwhelming media coverage, potentially leading to physical and psychological disorders [8]. As a notable emotional reaction, anxiety was a significant outcome of the COVID-19 pandemic, observed in healthy individuals, but with especially amplified consequences for those with pre-existing psychological vulnerabilities [10].

The COVID-19 pandemic meant people had to isolate and reduce contact, which was key to stopping the virus. But since socializing is vital for us, this isolation often leads to frustration and boredom. Studies show these restrictions harmed people’s mental well-being [11]. Also, fear grew when people felt they had no control, especially during an unexpected pandemic with changing advice [12]. Inconsistent messages from government authorities and healthcare representatives made people more insecure, confused, and scared [13].

The pandemic, with its limited interactions and economic troubles, will likely have lasting mental health consequences [14]. Several factors can influence the population’s mental health during such a crisis, including religiosity and sociodemographic variables like gender, age, education level, and marital status [2,15,16,17,18,19,20,21,22,23,24].

Religiosity, in particular, can be a significant factor, either improving or worsening mental health during the COVID-19 pandemic. One study showed that religious devotion increases life satisfaction by reducing depression levels [25]. Similarly, another study conducted among followers of Islam found that religious beliefs were negatively correlated with depression [26]. Additional studies support the association between religiosity and better mental health, with lower levels of depression and anxiety [27,28]. However, one study from Malaysia found that negative religious coping was positively associated with depression and anxiety [29]. Similar findings were reported by DeRossett et al., who observed higher levels of anxiety when individuals employed negative religious coping strategies [30]. These mixed findings underscore the complex role of religiosity in mental health during the pandemic, particularly highlighting the specific impact of different coping styles and the need for further investigation into moderating factors.

This study aimed to examine the impact of sociodemographic characteristics (age, sex, education level, and marital status) on mental health during the COVID-19 pandemic, with religiosity serving as a moderating factor. These specific sociodemographic variables were selected based on prior evidence linking them to heightened vulnerability for depression and anxiety during large-scale disasters and epidemics. Given the dual and often contradictory role of religiosity as a powerful psychosocial determinant—which influences coping mechanisms, help-seeking behavior, and access to community support—testing its precise moderating effect on these sociodemographic disparities is crucial for identifying vulnerable subgroups. Based on this rationale, we propose the following hypotheses, aimed at precisely testing these relationships: Hypothesis 1 predicts that higher age will be associated with a higher risk of depression and anxiety among participants. Hypothesis 2 posits that higher religiosity will be associated with lower levels of depression and anxiety. Finally, Hypothesis 3 suggests that religiosity will significantly moderate the relationship between sociodemographic characteristics and mental-health outcomes, operating primarily as a buffering protective factor against psychological distress.

## 2. Materials and Methods

### 2.1. Participants and Study Design

This research was organized as a cross-sectional study that was conducted during the COVID-19 pandemic from September 2022 to September 2023. Participants in the research were adult citizens of Croatia from the Virovitica-Podravina County.

Inclusion criteria for participation in the research were being of legal age (older than 18 years) and providing written consent to participate in the research. Refusal to provide consent or withdrawal during participation led to exclusion. All data collected in the questionnaire were completely anonymous and entered into a specially created database, which was backed up on a portable storage device for security. The research initially included 1295 participants; however, after reviewing the questionnaires and excluding those that were incomplete or improperly filled out, the final sample consisted of 1131 participants, who were divided into two groups. Specifically, the RC group comprised individuals with a medically documented positive PCR test for SARS-CoV-2 (indicating prior infection and clinical recovery), while the NRC group included participants with a consistently negative PCR result and no documented clinical history of COVID-19. This group assignment was verified through the participants’ medical records and corresponding PCR documentation (both at the time of recruitment and retrospectively, if available).

### 2.2. Instruments

The research was conducted using a questionnaire, specially designed for the purpose of this research. The aforementioned questionnaire consisted of questions regarding sociodemographic data, Beck Anxiety Inventory (BAI), Beck Depression Inventory (BDI), and the Saint Clare Strength of Religious Faith Questionnaire (SCSORF) [31,32,33]. Regarding sociodemographic data, the following information was collected: gender (male/female), age (in years), marital status (married/common-law partnership, single, divorced, widowed), and education level (completed elementary school, high school, undergraduate degree, graduate degree). The Beck Anxiety Inventory (BAI) consists of 21 items designed to measure generalized anxiety and differentiate anxiety symptoms from those of depression. Participants respond on a 4-point Likert scale (0 = not at all, 1 = mildly, 2 = moderately, 3 = severely). The total score ranges from 0 to 63, with higher scores indicating higher levels of anxiety [31]. The Beck Depression Inventory (BDI), comprising 21 items, is one of the most widely used instruments for measuring the severity of depression. Respondents answered on a 4-point Likert scale (0–3), with scores ranging from 0 to 63. Higher scores indicate more severe depression. Results are categorized as follows: no depression (0–11), mild depression (12–26), moderate depression (27–49), and severe depression (50–63) [32]. The strength of respondents’ religious beliefs was assessed using the Saint Clare Strength of Religious Faith Questionnaire (SCSORF) [33]. The SCSORF consists of 10 items, with responses given on a Likert scale (1 = strongly disagree, 2 = disagree, 3 = agree, 4 = strongly agree). Higher scores indicate a greater importance of religion in daily life [34]. Validated Croatian translations of BDI-II and BAI were used; prior psychometric validation in Croatian samples informed instrument selection.

### 2.3. Procedure

The research was conducted in family medicine clinics within the Primary Healthcare Center of Virovitica-Podravina County via questionnaire, specially designed for the purpose of this research. All participants were volunteers who met the inclusion criteria for the research. Prior to the start of the research, the participants confirmed their willingness to participate by signing an informed consent form. All data collected in the questionnaire were completely anonymous and entered into a specially created database, which was backed up on a portable storage device for security. All analyses were conducted at the group level, ensuring anonymity, with participants identified by a unique code. The data collected through these sociodemographic questions and instruments in our questionnaire enable a detailed analysis of the impact of sociodemographic factors on mental health, with a focus on anxiety, depression, and religiosity as key variables. The conduct of this research was approved by the Ethics Committee of the County Hospital Virovitica (date of approval: 25 August 2021, Class: 510-03/21-01/3744, Reg. No.: 2189-43-02/1-21-2 NM), Primary Healthcare Centre Virovitica-Podravina County (date of approval: 6 September 2021, Reg. No.: 2189-67/1-01-2376/2021) and the Institute of Public Health of Virovitica-Podravina County (date of approval: 3 September 2021, Reg. No.: 2189-47-05-21-540).

### 2.4. Statistical Analyses

Categorical data are presented using absolute and relative frequencies. Differences in categorical variables were tested by the Chi-square test and Fisher’s exact test. Differences in dependent categorical variables were tested with the McNemar–Bowker test. The normality of the distribution of continuous variables was tested with the Shapiro–Wilk test. Continuous data are described by measures of mean and dispersion depending on the normality of the distribution. Differences in continuous variables were tested with the Mann–Whitney U test (Hodges–Lehmann median difference and a 95% CI), the Kruskal–Wallis test, and the Friedman test between measurements (Post hoc Test Conover). The Bonferroni correction was used for multiple comparisons. All *p*-values were two-sided. The significance level was set at alpha (α) = 0.05. For statistical analysis, the statistical programs MedCalc^®^ Statistical Software version 22.018 [35] and SPSS 23.0 [36] were used. Results of Shapiro–Wilk tests are reported in the Results; where normality was rejected, nonparametric tests (Mann–Whitney U, Kruskal–Wallis, Friedman with post hoc Conover) were applied.

## 3. Results

### 3.1. Descriptive Indicators

The conducted cross-sectional research included 1131 respondents, divided into two groups. The first group consisted of 423 respondents who recovered from COVID-19 (RC), while the second group consisted of 708 respondents who did not recover from COVID-19 (NRC). Descriptive indicators were analyzed for the variables examined in the research, providing essential insight into the distribution and variability of the data. In the RC group, the average age was 56.51 years (SD = 18.40), with the majority of respondents being women (63.2%) and the remaining 36.8% men. Regarding marital status, the majority were married or cohabiting (66.0%). Most participants (52.3%) had completed high school. The average level of depression among these respondents was 8.92 (SD = 9.2), with scores ranging from 0 to 47, while the average level of anxiety was 11.49 (SD = 11.31), with scores ranging from 0 to 52. The average religiosity score was 28.78 (SD = 8.59) (Table 1).

In the NRC group, the average age was 53.08 years (SD = 17.60). The majority of respondents were women (61.40%). Regarding marital status, most participants were married or cohabiting (74.20%), and the majority (57.90%) had completed high school. The average level of depression in this group was 7.46 (SD = 8.37), with scores ranging from 0 to 52, while the average level of anxiety was 9.77 (SD = 10.82), with scores ranging from 0 to 59. The average religiosity score was 28.59 (SD = 8.68) (Table 1).

When comparing the descriptive indicators between the two groups (RC vs. NRC), no significant differences were observed across the test variables.

### 3.2. Correlation Analysis of Variables Examined in the Study

In reviewing the results of the correlation matrix presented in Table 2, which was constructed to explore the fundamental relationships between variables in participants in the RC group, we observe several significant associations. Age demonstrates a positive correlation with both depression (r = 0.46, *p* < 0.01) and anxiety (r = 0.40, *p* < 0.01). Additionally, a positive relationship is observed between age and religiosity (r = 0.21, *p* < 0.01), suggesting that older participants who have recovered from COVID-19 exhibit higher levels of religiosity. A moderate positive correlation is also observed between depression and anxiety (r = 0.76, *p* < 0.01), a finding consistent with the known comorbidity of these conditions. Religiosity shows weak but significant positive correlations with both depression (r = 0.10, *p* < 0.05) and anxiety (r = 0.20, *p* < 0.01).

In examining Pearson correlations among the variables of age, depression, anxiety, and religiosity in participants in the NRC group, several significant relationships are identified as well. Age is significantly positively correlated with depression (r = 0.42, *p* < 0.01), anxiety (r = 0.35, *p* < 0.01), and religiosity (r = 0.18, *p* < 0.01). Depression and anxiety display a strong positive correlation (r = 0.80, *p* < 0.01), underscoring the high comorbidity between these mental health conditions. Religiosity is also weakly but significantly correlated with anxiety (r = 0.08, *p* < 0.05).

It is important to highlight the differences in the strength of correlations between the participants in the RC and NRC groups. In the RC group, stronger correlations are observed between age and depression, anxiety, and religiosity, as well as between religiosity and anxiety. Notably, the relationship between religiosity and depression, which is significant in the RC group, is not significant in the NRC group. This pattern may suggest that recovery from COVID-19 has specific implications for mental health, potentially influencing the interrelationships between the variables studied.

Correlation analysis revealed a significant positive correlation between age and depression (r = 0.46, *p* < 0.01), as shown in Table 2, indicating that older participants who recovered from COVID-19 are more prone to depression.

To further validate the obtained results, a similar analysis was conducted on participants who had not recovered from COVID-19 (NRC group) to isolate the potential effect of recovery on this relationship. A significant positive correlation between age and depression (r = 0.42, *p* < 0.01) was also observed in this NRC group, although the strength of the correlation was slightly weaker. In both groups, the correlations fall within the range of moderate strength. However, the stronger association in the RC group suggests that the experience of recovery from COVID-19 may play a role in exacerbating depressive tendencies among older individuals. This relationship warrants further investigation.

Correlation analysis demonstrated a significant positive correlation between religiosity and depression (r = 0.10, *p* < 0.05), as well as between religiosity and anxiety (r = 0.20, *p* < 0.01), as shown in Table 2, indicating that more religious participants who recovered from COVID-19 (RC group) experienced higher, rather than lower, levels of depression and anxiety. This suggests an opposite trend to what was originally proposed.

As in the previous analysis, these findings were further examined by analyzing the relationship between religiosity and both depression and anxiety in participants in the NRC group. In this group, a significant but weaker positive correlation between religiosity and anxiety (r = 0.08, *p* < 0.05) was observed, while no significant correlation was found between religiosity and depression. This suggests that the association between religiosity and higher levels of depression and anxiety is specific to the RC group. These findings reveal an important distinction between the RC and NRC groups that merits further exploration.

To assess the moderating role of religiosity in the relationship between sociodemographic variables (age, gender, education, marital status) and mental health following recovery from COVID-19, interaction effects were analyzed, as shown in Table 3. Normality diagnostics (Shapiro–Wilk) for continuous variables are provided; given departures from normality, nonparametric tests were employed as specified. The obtained results indicated that religiosity significantly moderates the relationship between education and both depression (β = −0.50, *p* < 0.01) and anxiety (β = −0.69, *p* < 0.01). These findings suggested that participants with lower levels of education who are more religious tend to have better mental well-being—specifically, lower levels of depression and anxiety—compared to their less religious participants with similar sociodemographic profiles. These findings suggested that religiosity influences the relationship between education and mental health outcomes.

In addition to analyzing the moderator effect in participants in the RC group, the moderation effect was also tested in the NRC group. The obtained results revealed a significant interaction effect between age and religiosity in relation to depression (β = 0.12, *p* < 0.05), indicating that religiosity moderates the relationship between age and depression. In other words, the influence of age on depression differs based on religiosity levels. A similar pattern was found with respect to gender, where a significant interaction effect between gender and religiosity (β = 0.57, *p* < 0.01) suggests that the effect of gender on depression is moderated by religiosity. Additionally, a significant interaction effect was observed between marital status and religiosity (β = 0.54, *p* < 0.01), indicating that religiosity moderates the relationship between marital status and depression, meaning that the impact of marital status on depression varies depending on religiosity.

Moderator effects were also observed when anxiety was the criterion variable. A significant interaction effect between gender and religiosity (β = 0.52, *p* < 0.05) suggests that religiosity moderates the relationship between gender and anxiety. Furthermore, a significant interaction effect between marital status and religiosity (β = 0.43, *p* < 0.01) was found, indicating that religiosity moderates the relationship between marital status and anxiety.

Among participants in the RC group, religiosity significantly moderated the relationship between education and mental health, with lower education levels in combination with high religiosity being associated with lower levels of depression and anxiety. In contrast, among participants in the NRC group, religiosity significantly moderated the relationships between age, gender, marital status, and mental health. Specifically, older, male, and married participants with higher levels of religiosity exhibited lower levels of depression and anxiety. These findings suggest that religiosity plays different protective roles depending on the health context: among individuals who recovered from COVID-19 (RC group), it appears to benefit those with lower levels of education, while among those who did not recover (NRC group), religiosity provides support across a broader range of sociodemographic groups.

This highlights the complex role religiosity plays in mental health and underscores the importance of tailoring mental health interventions to meet the needs of diverse groups.

## 4. Discussion

The first hypothesis, which proposed that older individuals would exhibit a greater tendency toward depression after recovering from COVID-19 compared to younger individuals, was confirmed. Partially aligning with the findings of this research, a study conducted by the Consultation Multi-Expertise de Bicêtre Après COVID-19 (COMEBAC) group found that individuals over the age of 75 who had been infected with COVID-19 exhibited more symptoms of depression compared to those under 75 years old [21]. Similar results were reported by another study, which demonstrated higher levels of depression among individuals above 65 who had been infected with COVID-19 [15]. Partially supporting these findings, age was identified as a potential factor in the relationship between depression and COVID-19 infection [37]. Conversely, some studies have yielded conflicting results. For instance, one study reported that younger individuals who were infected with COVID-19 were at a higher risk of developing depression [38], while another one found that the same population group exhibited higher levels of depression [2]. According to these authors, the reduced mental health impact of social isolation on older adults, when compared to younger individuals, could be attributed to their pre-existing lower social activity levels. Additionally, older adults may have developed greater resilience due to their prior experiences with other life-threatening circumstances, such as previous pandemics and epidemics.

The inconsistencies in the results of previous studies indicate that age may not uniformly predict the risk of depression following COVID-19 infection. However, it is reasonable to conclude that older adults represent a high-risk group for developing depression as a result of the COVID-19 pandemic, particularly in cases of infection. This population was consistently identified in the media and by experts as being at elevated risk for infection. Moreover, severe cases of the disease and fatal outcomes were more prevalent among older adults, which likely heightened their fear of infection and contributed to the development of depressive symptoms.

Social isolation, which was particularly recommended for the elderly during the pandemic, may have further contributed to the onset of depression. Reduced social contact, limited interaction with family and friends, and increased feelings of loneliness are all potential factors that could lead to depression in this population. Given these findings, it is clear that older adults are a vulnerable group in terms of mental health, and targeted interventions should be implemented to provide adequate support and prevent the development of mental health difficulties in this population.

Our results did not support the second hypothesis, which proposed that more religious participants would exhibit lower levels of depression and anxiety after recovering from COVID-19. Contrary to expectations, the data analysis indicated that participants with higher religiosity who recovered from COVID-19 exhibited elevated levels of depression and anxiety. This finding is inconsistent with prior research. For instance, greater religious devotion during the pandemic was found to increase life satisfaction by reducing depressive symptoms, a result consistent with the original hypothesis [25]. Similarly, individuals with stronger Islamic faith had lower levels of depression during the pandemic [26]. Positive religious coping strategies among Islamic adherents also reduced symptoms of depression [28], while intrinsic religiosity and belief in God were associated with lower anxiety levels during the pandemic [27].

However, partial alignment with the current study’s findings can be seen in another study, which indicated that more frequent use of both positive and negative religious coping strategies correlated with heightened anxiety about mortality [39]. Similarly, negative religious coping was also positively associated with increased anxiety levels related to the COVID-19 pandemic [30].

The present findings suggest that religiosity did not always serve a protective function during the COVID-19 pandemic, but in certain individuals may have intensified psychological distress through negative religious coping. This form of coping, described in the literature, includes feelings of guilt, anger toward God, or perceiving illness as divine punishment [40]. It is possible that participants with higher religiosity who recovered from COVID-19 experienced internal conflict, questioning their faith or interpreting their illness as a moral or spiritual failure. Such cognitive and emotional dissonance can contribute to higher depression and anxiety levels. These patterns align with prior studies reporting that negative religious coping during health crises is linked to worse psychological outcomes [41].

This psychological struggle, sometimes referred to as ‘spiritual discontent,’ is particularly relevant in pandemics, when individuals may seek meaning in suffering but instead experience spiritual disappointment. In these situations, religiosity can function as a negative factor by reinforcing guilt or self-blame, rather than providing comfort. Our data suggest that this mechanism may partly explain the observed association between higher religiosity and increased depression and anxiety levels among participants who recovered from COVID-19.

Thus, it is crucial to recognize the type of religious coping strategies being utilized during such challenging periods.

From a public health perspective, these findings underscore the need for collaboration between mental health services and faith-based organizations to provide education on adaptive religious coping. Integrating spiritual counseling or pastoral care within psychological support frameworks could help individuals use their faith as a source of resilience rather than distress. Training community leaders and clergy in recognizing signs of spiritual struggle could also facilitate early identification of individuals at risk for psychological distress, thereby reducing the negative mental health impact of future crises.

The third hypothesis, which proposed that religiosity moderates the relationship between sociodemographic variables (age, gender, education, and marital status) and mental health after COVID-19, was partially confirmed. The findings revealed that religiosity moderates the relationship between education and both depression and anxiety, with participants who have lower levels of education but higher levels of religiosity showing reduced depression and anxiety. Although this study did not establish religiosity as a moderating factor between gender and mental health following COVID-19 recovery, other studies have highlighted gender differences in religiosity, religious coping styles, and mental health during the pandemic.

For example, gender differences in coping strategies during the COVID-19 pandemic were found, with women being more likely to employ various coping strategies, particularly religious ones [42]. These results persisted even after controlling for variables such as education level, household income, family structure, and marital status. Similarly, female students were more likely to use religious coping strategies compared to male students, and women were identified as a higher-risk group for anxiety during the pandemic [43].

Male participants with lower income and incomplete family structures were more inclined to use negative religious coping strategies, although nearly all adolescents reported anxiety related to the pandemic [44]. Women were more likely to experience fear and anxiety about the possibility of death due to COVID-19 infection, while also reporting a strengthening of their religiosity during the pandemic [45].

In contrast to these findings, one study of quarantined elderly individuals found that higher levels of depression, anxiety, and stress were associated with being female, but not with age or religiosity [46]. Furthermore, among participants of the Islamic faith, religious coping was negatively associated with depressive symptoms, and this relationship was not moderated by sociodemographic factors such as age, gender, or education level [28].

One Italian study showed that younger participants reported lower levels of spiritual well-being and faith, which were associated with poorer mental health outcomes [47]. A study using representative data from the United States aimed at exploring age differences in mental health problems during the COVID-19 pandemic, with depression and anxiety as focal variables [48]. This research confirmed the moderating role of attachment to God in the relationship between age and mental health problems. Specifically, older participants (61 years and older) were more likely to experience lower levels of depression and anxiety, but this difference diminished when participants had a secure attachment to God, suggesting that such attachment contributed to lower levels of depression and anxiety across age groups.

When considering the relationship between education level, religiosity, and mental health, a study revealed that within a Christian sample, individuals with lower education reported a higher prevalence of psychological disorders [28]. However, a study of 1250 adults during the first period of isolation in Italy found no significant differences in spiritual well-being or mental health concerning education level [47]. Additionally, a meta-analysis highlighted an increase in anxiety during the COVID-19 pandemic, identifying younger age, lower education, and marital status as significant risk factors [49].

Prior research on sociodemographic factors and mental health has yielded inconsistent results. Nevertheless, existing evidence suggests that women and individuals with lower educational levels face a greater risk for mental health issues. Religiosity typically appears to offer a protective effect against depression and anxiety, particularly in younger individuals.

However, the present study revealed a somewhat contradictory pattern where participants with lower education but lower levels of religiosity reported better mental health outcomes. This may indicate that, in certain cases, higher religiosity could be associated with increased psychological distress due to the use of negative religious coping. Such coping includes feelings of guilt, anger toward God, or interpreting illness as divine punishment, which have been shown to elevate distress during crises [40]. It is plausible that some participants viewed COVID-19 infection as a test of faith or a form of divine retribution, amplifying negative emotions and impeding recovery. Prior research confirms that “spiritual struggle” and “divine discontent” predict higher anxiety and depressive symptoms [41]. Conversely, positive religious coping trust in God, prayer as comfort, and finding meaning in suffering, has protective effects on mental health [50]. These findings emphasize the importance of differentiating between positive and negative religiosity when analyzing psychological outcomes in times of crisis.

It is possible that individuals with lower levels of education were less informed about the COVID-19 virus and may have had a poorer understanding of the recommendations made by health officials, doctors, and other experts. There is also uncertainty about whether individuals with lower education could critically assess the media coverage surrounding the pandemic, which often conveyed alarming information. This could have led to heightened fear among less educated individuals, particularly if they became infected, contributing to increased levels of anxiety and depression. Nonetheless, religion may have provided hope for recovery, faith in better days ahead, and emotional support through prayer, particularly for individuals with lower education. In such cases, faith may have served as a calming force, improving mental well-being and ultimately leading to lower levels of depression and stress.

From a public health perspective, these findings also have important implications. During crises such as pandemics, cooperation between public health institutions and faith communities can be crucial in mitigating psychological distress. Integrating spiritual counseling and pastoral care into mental health services, along with education on adaptive religious coping strategies, may help reduce maladaptive emotional responses and enhance resilience. Public health systems that encourage community-based, compassion-focused faith practices such as collective prayer, meaning-making, and social support can strengthen overall population well-being [51].

### 4.1. Limitations of the Study

We acknowledge several limitations of our research. This study relied on self-report instruments, sampled a single county with group size imbalance, and employed a cross-sectional design that precludes causal inference; residual confounding cannot be excluded. Therefore, future research should be conducted across multiple countries using a multicentric approach, including questions on religious affiliation and practice, and ensuring a more balanced number of respondents in each group. Additionally, future research would benefit from comparing vulnerable participants as one group with the general population as a second (control) group, both of whom had a COVID-19 infection.

### 4.2. Public-Health Implications

Integrating culturally sensitive spiritual support within mental-health services and partnering with faith-based organizations may improve outreach and outcomes among vulnerable subgroups during future crises.

## 5. Conclusions

According to WHO guidelines, it is essential to reduce and control the incidence of COVID-19 infection, as well as to diagnose and treat its long-term consequences. Protecting individuals, especially vulnerable groups, from COVID-19 infection is crucial, along with preparing the healthcare system for future outbreaks.

This study demonstrated that sociodemographic characteristics are significant predictors of mental health during the COVID-19 pandemic. Religiosity emerged as an important factor, particularly in moderating the relationship between education and mental health among participants who recovered from COVID-19, as well as moderating the relationship between age, gender, and marital status in the control group. These findings underscore the necessity of providing social and spiritual support, along with the development of targeted interventions aimed at improving mental health outcomes.

## Figures and Tables

**Table 1:** Descriptive Indicators of Variables.

Variable	Number of Participants	Minimum	Maximum	Arithmetic Mean	Standard Deviation
Age	423 (RC) 708 (NRC)	18 (RC) 18 (NRC)	89 (RC) 92 (NRC)	56.14 (RC) 53.08 (NRC)	18.23 (RC) 17.60 (NRC)
Depression	423 (RC) 708 (NRC)	0 (RC) 0 (NRC)	47 (RC) 52 (NRC)	8.92 (RC) 7.46 (NRC)	9.22 (RC) 8.38 (NRC)
Anxiety	423 (RC) 708 (NRC)	0 (RC) 0 (NRC)	52 (RC) 59 (NRC)	11.49 (RC) 9.77 (NRC)	11.31 (RC) 10.82 (NRC)
Religiosity	423 (RC) 708 (NRC)	5 (RC) 10 (NRC)	45 (RC) 40 (NRC)	28.78 (RC) 28.59 (NRC)	8.59 (RC) 8.87 (NRC)

RC = Recovered from COVID-19, NRC = Not Recovered from COVID-19.

**Table 2:** Correlation Matrix of Variables Examined in the Study.

	Age	Depression	Anxiety	Religiosity
Age	1	0.46 ** (RC) 0.42 ** (NRC)	0.40 ** (RC) 0.35 ** (NRC)	0.21 ** (RC) 0.18 ** (NRC)
Depression		1	0.76 ** (RC) 0.80 ** (NRC)	0.10 * (RC) 0.03 (NRC)
Anxiety			1	0.20 ** (RC) 0.08 * (NRC)
Religiosity				1

* = *p* < 0.05, ** = *p* < 0.01, RC = Recovered from COVID-19, NRC = Not Recovered from COVID-19.

**Table 3:** Beta Coefficients and Their Significance in Regression Analyses with Religiosity as a Moderator, Using Depression and Anxiety as Outcome Variables.

Predictor and Moderator	Criterion	β
Education × Religiosity	Depression	−0.50 **
Education × Religiosity	Anxiety	−0.69 **
Age × Religiosity	Depression	0.12 *
Gender × Religiosity	Depression	0.57 **
Marital Status × Religiosity	Depression	0.54 **
Gender × Religiosity	Anxiety	0.52 *
Marital Status × Religiosity	Anxiety	0.43 **

* = *p* < 0.05, ** = *p* < 0.01.

## Data Availability

All data are available and can be delivered to anyone upon request. The data presented in this study are available on request from the corresponding author due to privacy or ethical restrictions.
